# Supplementary material for: Framing South Asian politics: An analysis of Indian and Pakistani English print media discourses regarding Kartarpur corridor
Source: PLoS One. 2022 Feb 17;17(2):e0264115. doi: 10.1371/journal.pone.0264115 (PMC8853468; doi:10.1371/journal.pone.0264115)
Supplement: Attachment — Submitted filename: A. Response to Reviewers.docx [file pone.0264115.s001.docx]

**Response to Reviewers’ Comments**

**English editing is required by a native English speaker (please provide with certificate of editing from a recognized agency/editing service).**

The article has been thoroughly proofread by the authors. In addition, it was also edited by the language expert. The editing certificate is attached as proof.

**Write up is loaded with lot of issues right from abstract to conclusions. Please revise almost all sections of the paper for clarity, conciseness, and novelty.**

Thank you for highlighting this issue, all the sections of the paper have been revised and made the changes by deleting extra information and adding only relevant information concisely.

**Explain clearly the novelty of the paper in terms of scientific research gap.**

This paper is carried out by applying the lexical study of Natural Language Processing (NLP) through its Latent Dirichlet Allocation (LDA) tool to find out the general patterns in the print media of both countries. LDA provides several keywords and arranges them based on their weightage in the group. The word having high weightage refers to the influence of that word in the group. In addition detailed research gap has been provided (see page 4, line 3-19)

**A reasonable rationale is missing. Problem statement could be more convincing and obvious.**

After reviewing the previous literature, it is noted that no concrete corpus-based research has thus far been carried out using NLP techniques on the newspaper's data and media framing through the respective national print media regarding the Kartarpur corridor. Therefore, it becomes quite important to investigate how the issue of Kartarpur has been echoed on both sides of the border, which took more than 70 years to open as the media played a key role in framing the concerns and opportunities in the masses on both sides.

**Try not to use bullet points in the introduction section or anywhere until those are unavoidable.**

Thanks for pointing out; however, we thought, discussed and kept as it is to align with the previous discussion of the section to give clearer picture of the review

**Try not to make new section/sub-section if that consists of only 1 or 2 lines.**

Thanks for pointing out the issue. The subsections have been merged under one section especially in methodology section (please see page 4. Line 20-48, page 5 line 27-49)

**Methodology is lengthy and unorthodox. Please follow a standard version of methodology and explain study area, data collection, sampling, methods and analysis clearly one by one.**

Done accordingly by removing irrelevant and adding relevant material.

**Current presentation of figures and tables is too poor to grasp. Few of them are screenshots. Captions do not present complete information and formatting is awful. Adjust all of these issues. Provide sources of the figures and tables under the caption as it is done in scientific papers.**

Very comprehensive feedback. We have addressed all these issues accordingly. Moreover, we have conducted analysis using Python programming language. The results, produced through Python programming language have been exported in the form of images. This is the reason that all results are in the form of images. However, the quality of images has been improved.

**Increase the canvas of analysis and provide more information in the form of tables and figures. Two tables and figures are insufficient.**

[Each table has been explained thoroughly. Added new figures with the explanation

**Third section is data analysis and 4th was supposed to be results. How can authors discuss results without presenting results? This is a blunder.**

[Changed accordingly as Interpretation of Results with Analysis]

**How about conclusions sections? Without conclusions how one can reach to the study's findings?**

[Revised the conclusion section comprehensively]

**Additional Changes**

The manuscript has been formatted according to journal template guidelines.
