# Supplementary material for: Framing South Asian politics: An analysis of Indian and Pakistani English print media discourses regarding Kartarpur corridor
Source: PLoS One. 2022 Feb 17;17(2):e0264115. doi: 10.1371/journal.pone.0264115 (PMC8853468; doi:10.1371/journal.pone.0264115)
Supplement: Attachment — Submitted filename: Response to Reviewers.docx [file pone.0264115.s002.docx]

**Response to Reviewers**

**Reviewer’s Comments:** A comprehensive interpretation of results based on figures is missing. The authors should fill this gap.

**Authors’ Response:** As per the recommendations of one the reviewers, the details about Figures has been incorporated (Please see page 9, 10, 11 and 12). Figures have been discussed in detail.

**Reviewer’s Comments:** The conclusion section is too short. Although the authors described the two major concluding points in this section. However, based on the scope of this article, results and discussion still there exist room for improvement.

**Authors’ Response:** The conclusion section has been enlarged by adding one more paragraph based on the results and discussion.

**Additional Changes:**

Some typos have been corrected throughout the manuscript. The track changes version is available as proof.
